# Algal solutions: Transforming marine aquaculture from the bottom up for a sustainable future

**DOI:** 10.1371/journal.pbio.3001824

**Published:** 2022-10-17

**Authors:** Charles H. Greene, Celina M. Scott-Buechler

**Affiliations:** 1 Friday Harbor Laboratories, University of Washington, Friday Harbor, Washington, United States of America; 2 Department of Earth and Atmospheric Sciences, Cornell University, Ithaca, New York, United States of America; 3 School of Earth, Energy & Environmental Sciences, Stanford University, Palo Alto, California, United States of America; Brown University, UNITED STATES

## Abstract

Current aquaculture practices are unsustainable. In this Perspective, the authors argue that shifting the focus of marine aquaculture down the food chain to microalgae could meet projected global nutritional demands while simultaneously protecting ocean health.

## Teaser

Today’s global food production system is unsustainable. Shifting the focus of marine aquaculture down the food chain to algae could help meet projected global nutritional demands while simultaneously improving overall environmental sustainability and ocean health.

With the world’s population approaching 10 billion people by 2050, global food production will need to increase by >50% to meet humanity’s projected nutritional demand [1]. Currently, terrestrial agriculture provides the backbone of the global food production system. However, agriculture’s potential to close the projected mid-century nutritional gap will be constrained by its negative impacts on climate, land use, freshwater resources, and biodiversity [2]. If we turn to the ocean to try to close this nutritional gap, then we are immediately confronted with the realization that most wild-capture fisheries are already fully exploited or overexploited [3].

A variety of alternative food options are being explored to determine how society might sustainably intensify its food production system. Among these, the cultivation of finfish, shellfish, and seaweeds in nearshore aquaculture facilities has attracted much attention [4]. However, even a vast expansion of current aquaculture practices around the world would run out of available space in the coastal ocean before closing the projected nutritional gap [2]. In addition, such an expansion would likely pose significant environmental threats, such as increasing the eutrophication of coastal waters and accelerating the spread of pathogens and parasites among wild populations. It would also lead to conflicts with the many other stakeholders vying for use of the coastal ocean.

**Fig 1:**
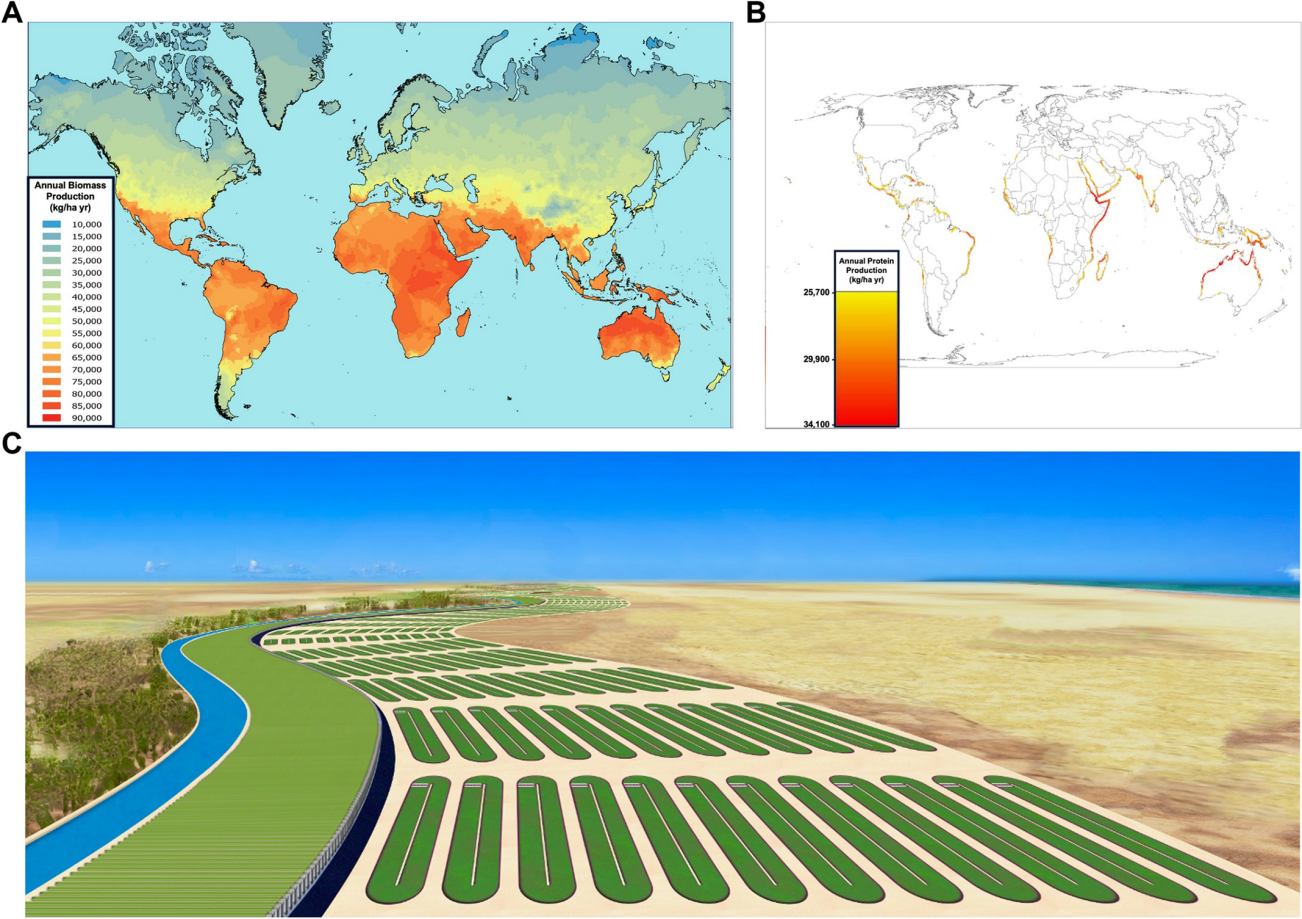
Opportunities for a global marine microalgae aquaculture industry. (A) Global map of onshore microalgae biomass production potential based on solar insolation data and an algal growth model, as modified from [2]. Solar insolation limits higher productivity potential to lower latitudes. (B) Global map of onshore microalgae protein production potential constrained by various environmental and financial considerations, as modified from [2]. Inexpensive access to seawater limits marine microalgae aquaculture facilities to a relatively narrow band along coastlines. (C) Illustration of marine microalgae aquaculture facilities located along a coastal desert plain, modified from [5].

One potential solution to the food production problem that could have both nutritional and environmental sustainability advantages would be to shift the focus of aquaculture down the marine food chain to microalgae. As a large polyphyletic group with many unstudied species, microalgae offer a potentially substantial, mostly untapped source of high-quality nutrition [2]. With many species possessing protein contents exceeding 40% of their dry mass, microalgae typically provide a better source of nutritional protein and essential amino acids than terrestrial plants. In addition, they provide certain micronutrients, including vitamins, antioxidants, omega-3 polyunsaturated fatty acids, and minerals, that are difficult to derive from other nutritional sources. Similar to soy, microalgae-derived protein powders can be incorporated into the supply chains for producing dairy and meat substitutes as well as pastas and baked goods. Most importantly, because of their high productivity, typically 1 to 2 orders of magnitude greater than terrestrial plants, marine microalgae cultivated in onshore aquaculture facilities have the potential to meet all of the global protein demand projected for 2050 [2,5] (Fig 1).

In terms of overall environmental sustainability, because the onshore cultivation of marine microalgae does not require soil, irrigation, and fertilizer application in the open environment, a marine microalgae-based aquaculture industry would not need to compete with agriculture for arable land and freshwater nor lead to fertilizer runoff and the subsequent eutrophication of freshwater and marine ecosystems [2,5]. Furthermore, by reducing agriculture’s arable land and freshwater footprints, such an aquaculture industry could reduce the pressure for deforestation, especially in the tropical rainforests of Southeast Asia and the Amazon, potentially leading to globally significant reductions in greenhouse gas emissions and terrestrial biodiversity loss [2].

A marine microalgae-based aquaculture industry also has the potential to reduce some of the greatest threats to ocean health. Anthropogenic carbon dioxide (CO_2_) emissions pose the greatest long-term threat to global ocean ecosystems [6]. Greenhouse warming is increasing the intensity, frequency, and duration of marine heat waves in the upper ocean. In turn, these marine heat waves are leading to coral bleaching, marine disease outbreaks, and species’ range shifts. Deeper in the ocean, warming temperatures are leading to deoxygenation and the expansion of oxygen minimum zones, both of which are detrimental to organisms with aerobic metabolisms. And finally, the rapid rise in atmospheric CO_2_ concentration is driving ocean acidification, which currently is having its greatest impacts in colder, high-latitude marine ecosystems, but will be impacting all upper ocean ecosystems by the end of the 21st century.

As part of an emerging marine circular bioeconomy [2], the development of a marine microalgae-based aquaculture industry can diminish these threats by reducing CO_2_ emissions and enhancing CO_2_ removal efforts. The land-use changes arising from shifting a portion of our food production system from terrestrial agriculture to microalgae-based aquaculture could lower global CO_2_ emissions significantly [2]. In addition, emissions could be further reduced by using microalgae to produce fuels and other products currently derived from petroleum [5].

However, to receive the full benefits of such emission reductions, it will be critical that the CO_2_ utilized in the cultivation process is derived from the atmosphere and not from fossil sources. This is not as simple as it sounds. When growing at optimal rates, microalgae take up ionic forms of CO_2_ more rapidly than they can be replaced by the diffusion of CO_2_ gas across the air–water interface of open cultivation systems. This means that CO_2_ must be added regardless of whether the cultivation system consists of open ponds or closed photobioreactors. Presently, CO_2_ is more readily available and less expensive when derived from fossil sources. Innovation will be required to make atmospherically derived CO_2_ an economically viable alternative.

Several studies have suggested that deriving the necessary CO_2_ could be achieved by integrating microalgae cultivation facilities with direct air capture (DAC) [5,7] or bioenergy with carbon capture and storage technologies [8]. Current DAC approaches are prohibitively expensive for this purpose; however, integrating DAC with concentrated solar power or other emerging renewable energy technologies could provide a cost-effective approach for simultaneously producing high-value commodity products, generating power, and capturing CO_2_ [9].

Furthermore, a marine microalgae-based aquaculture industry could improve the sustainability of wild-capture fisheries. Not only could such an industry reduce the pressure to overexploit wild stocks for human consumption, but also it could reduce the demand for fishmeal and fish oil used in aquafeeds for today’s finfish- and shellfish-dominated aquaculture industry. The production of fishmeal and fish oil replacement products from marine microalgae could potentially reduce the global wild-capture fisheries harvest by up to 30% and generate a global market value approaching $6.5 billion in annual net income [10]. That potential aquafeed income would correspond to 2.6% of today’s global seafood industry’s market value, demonstrating that marine microalgae-based aquaculture could become good for business as well as for human nutrition and ocean health.

Regardless of its potential nutritional and environmental benefits, a marine microalgae-based aquaculture industry will need to challenge incumbent industries in the food sector for market share. The financial headwinds faced by this new industry will be stiff because it must meet this challenge before its technologies are completely mature and before it can achieve the full benefits of scale. On the positive side, these are the same green premium challenges faced and overcome by the solar and wind energy industries in their early days. Financial investments and market incentives provided by state and federal governments can help reduce this green premium until the playing field is level. The future role of algae-based solutions in achieving global food security and environmental sustainability will depend on the actions taken by governments today.
